# Dr. Adams, on Consumption

**Published:** 1801-11

**Authors:** 


### *Dr.* ADAMS, *on Consumption.*

DR. ADAMS's Correfpondent informs us, that he has received another letter from that gentleman, in which he very much enforces the objection he made before, relative to fending patients late in the difeafe to Madeira. It muft be admitted that the fault is more commonly in the patient, or his friends, than in the phyfician, who has frequently advifed the voyage feveral months before he could prevail on the patient to undertake it. This delay often proves fatal; and what much increafes the diftrefs of a family is the lofs of a relation at a diftance from home. All this is well known to the Faculty, and perhaps fuch cautions might be altogether unneceffary in a work intended entirely for medical readers. One general caution, however, he cannot help enforcing: If a patient has been lofing ground all the winter, with tolerable management he may do as well during the fummer in England as in moft other parts of the world; if he has grown worfe during the fummer, he is not likely to improve by any removal to a warm climate: But if he has found a refpite during the fummer, for complaints exafperated during a former winter, or if at any period of the winter he is threatened with fymptoms which render a mild and fettled climate defirable, Dr. A. fcruples not to fpeak of Madeira as in his former letter; having feen many releafed from a ftate of the difeafe which in England proves invariably fatal in the courfe of the winter.

Thefe remarks may be extended to all thofe pulmonary difeafes which are exafperated by a winter, whether *Tussis Senilis*, which is called the Humoral Afthma, as well as all fcrofulous complaints.

*Madeira, Sept.* 17, 1801.

